# Bristol Medico-Chirurgical Society

**Published:** 1899-06

**Authors:** J. Paul Bush

**Affiliations:** Hon. Sec.


### MEETINGS OF SOCIETIES.

### Bristol Medico-Chirurgical Society.

*March 8th*, 1899.

Dr. R. Roxburgh, President, in the Chair.

Dr. H. Waldo showed (1) a boy, aged 11 years, with Adenoma sebaceum of the face, which had existed for about a year. He said that this disease was characterised by the appearance of neoplasms papular in character, of congenital origin, but appearing at or before puberty. There is a distinct outline to each lesion, and coalescence is unusual. A large proportion of the subjects are epileptics or imbeciles, and it was at one time supposed that bromides produced the eruption. Adenoid changes are found in the sebaceous glands. The papillary vessels are conspicuous, and there is an increase in the connective tissue. There is, in fact, a replacement of large numbers of hair-follicles and sebaceous glands by fibrous tissue, of which the little tumours are largely composed. There is extensive interpapillary hypertrophy. The lesions may be flat-topped, others rounded, convex or acuminate, while some may be warty-looking, and may be pedunculated, though the customary papule is sessile. Involution may occur, but the tendency is to persistence, the lesions recurring in spite of removal and even *in situ*. (2) A young woman with tuberculosis of the skin. The patient received a wound of the right leg six months ago. She says that shortly after this a rash extended all over her. When first seen there was a broad streak of lupus erythematosus extending down the left arm and forearm, with a circular patch the size of a half-crown on the right forearm, and also a well-marked bat's wing arrangement on the face and nose, with a slight implication of the left ear; in fact, the disease was becoming generalised. This condition had been present for four months. She thought she had lost flesh. There was no cough. He prescribed an emollient and slightly astringent lotion during the day, and an ointment of sulphur and carbolic acid to be rubbed in at night. She was to take cod-liver oil and quinine; to keep her bedroom window well open at night, and to be in the open air as much as possible in the day. The patient was now nearly well.

Dr. J. E. Shaw showed (1) a case of acute anterior poliomyelitis, which had existed six months, involving the whole of the muscles of both legs, including the glutei; the case is remarkable in that not the slightest degree of recovery has taken place. (2) A case of spastic diplegia which began to develop at 9 months of age. Patient now has the usual cross-kneed progression, but the legs are permanently bent upon the thighs; the feet are long, flat, not in a position of talipes equinus, and are everted. (3) Two cases of pseudo-hypertrophic muscular paralysis occurring in brothers. The younger, aged 6, exhibits the usual phenomena of an early stage, being able to stand and walk alone and rise from the floor unaided; the knee-jerk is present. The elder, aged 12, cannot stand or walk alone, and cannot rise from the ground beyond getting on to his knees. He stands, when sustained artificially, upon his toes and toe-balls, the heel never coming down upon the ground owing to the contraction of the calf muscles maintaining the feet in the position of talipes equinus. The calf muscles are not very large, but are very hard; the knee-jerk is absent on both sides.

Dr. Michell Clarke showed (1) a case of syringomyelia in a young man of 17. The illness began in July, 1898, with weakness and wasting of the muscles of the left hand. There had been no pain throughout. The symptoms present now are: atrophy of the small muscles of the left hand and of extensors of the left forearm, with paralysis of the left upper extremity; wasting to a less degree of the left deltoid, biceps, pectoralis major, and of the small muscles of the right hand; loss of sensation to pain, and to heat and cold, but not to touch, over left arm and left side of chest. There were also spastic rigidity of the legs, double ankle-clonus, left rectus-clonus, and exaggerated right knee-jerk. (2) A case of disseminated sclerosis in a sailor, aged 39, who had not had syphilis, and had enjoyed very good health. He attributed his illness to privation and exposure.

There were well-marked intention tremor of both arms, most in the right; scanning speech, exaggerated knee-jerks, weakness of arms and legs, attacks of vertigo, with defect of associated movement of the eyeballs to the left. (3) A case of progressive muscular atrophy with absent knee-jerks, and remarkably extensive distribution of the muscular atrophy for the presumably early stage of the disease.—Dr. WALDO asked Dr. Clarke if the retinæ had been examined ophthalmoscopically, as he considered that atrophy of the papilla was very often found in cases of disseminated sclerosis, and might assist in the diagnosis of an obscure case.—Dr. CLARKE, in reply, said that the optic discs were pale.

Dr. E. C. WILLIAMS showed a case of hemiplegia, with wasting of the muscles of the leg, of six years' duration in a child aged 11 years. The onset was sudden—pain over the left temporal region, followed by a convulsion. After the convulsion, there was loss of speech and paralysis of right side of face, right arm, and right leg. She gradually recovered from the aphasia and facial paralysis in about fourteen months. There was no history of rheumatism, chorea, or any infectious disease. She is of slow intellect and very forgetful. On admission to the Children's Hospital, under Dr. Lees, her right arm was in a spastic condition—elbow and wrist more or less flexed, fingers flexed or extended and rigid, movements being slow and usually tremulous. The right arm measures more than the left, the increase being probably due to the constant choreiform movements. Electrical reactions, normal; heart and lungs, healthy. Right leg measures 1 inch less than left. There is pes cavus. Some of the leg muscles give the reaction of degeneration; knee-jerks, glib. The seat of the lesion appears to be in the upper motor segment, for the following reasons: 1, The lesion is a hemiplegia; 2, aphasia and facial paralysis at onset; 3, no reaction of degeneration in upper limb; 4, reflexes present. The lesion is double; *i.e.*, there is an anterior poliomyelitis as well as an upper segment lesion (polio-encephalitis). It would appear the two lesions are the result of the same poison, which more commonly affects the anterior horns, but also affects the ganglion cells of the cortex. The disease is often epidemic, many cases being reported in the *Journal of the American Medical Association*, xxvi., 1896, in which the characteristic symptom was paralysis in young children. It was attributed to insanitary surroundings. An elder brother of this child had a similar attack seventeen years ago, but made a fair recovery.

Mr. MUNRO SMITH showed a boy on whom erasion of the knee-joint had been performed, a year ago, for extensive disease of the synovial membrane and cartilage. The bone was slightly affected. Two operations were performed before the disease was eradicated. The boy can now walk easily and well, and there is very slight shortening.

Mr. C. A. MORTON showed a woman, aged 59, from whom, in November last, he had excised the cæcum for a carcinomatous growth. The ileum was joined to the ascending colon with a Murphy button. The growth had been so movable that it simulated movable kidney. Not only could it be pushed into the loin, but also as far inwards as the umbilicus. The patient had gained 2 stone in weight since the operation.—Mr. ROGER WILLIAMS said cancer of cæcum was such a comparatively rare disease that but few examples of its successful removal have been reported. He could at that moment recall only two cases in which an operation of this kind had been done. One was by Homans, in which he successfully excised a sarcoma of the cæcum from a girl of 5. In another case Abbe excised the cæcum and several feet of intestine from a youth of 16, uniting the divided ends of gut by a Murphy button. The patient died shortly after operation. It was interesting that in Mr. Morton's case there were no symptoms of obstruction, as this had been noted in several cases of cæcal cancer, wherein it differed so markedly from other cancers of large intestine. Of 100 consecutive cases of cancer of the large intestine, Mr. Williams found that ·5 per cent. were cæcal and ·5 per cent. of ileo-cæcal valve, whereas Fagge found 4 per cent. were cæcal.—Dr. Lacy Firth remarked that one of the difficulties in the operation of removal of the cæcum was due to the difference in size of the two portions of bowel which had to be united. He had heard of one successful case, in which the end of the colon was partially closed by sutures and the small intestine then fixed to the portion remaining open by means of a Murphy button. He had on one occasion removed the cæcum for malignant disease, and had attempted to overcome the difficulty mentioned by closing up the end of the colon and then fixing the end of the ileum to its side with a Murphy button, but the patient unfortunately died on the seventh day. He asked if Mr. Morton had found any difficulty in his case in approximating the ileum to the colon by reason of the difference in calibre.—In reply to Mr. Roger Williams and Dr. Firth, Mr. Morton said he had not yet had time to look up the literature of the subject, but would refer to it when he published the case. There was considerable difficulty in getting the cut edge of the ascending colon into the grip of the button, which was the one used for the junction of small intestine.

Mr. T. Carwardine showed two patients after excision of the rectum for malignant disease by a method which he had devised and described in the *British Medical Journal*, 1898. Each patient has a partially renewed and bifid coccyx and intact sphincter, and perfect control over fæces, and the case operated on six months ago has control of flatus also. Defæcation is performed voluntarily once or twice a day, and rectal sense is perfect.—Mr. C. A. Morton asked if Mr. Carwardine left a segment of the lower end of the rectum in his two operations. He thought that if the growth extended far up the rectum the peritoneum must of necessity be opened in the removal of the growth, as in the ordinary Kraske's operation.—Mr. Carwardine, in reply, said the amount of anal mucous membrane left may vary with the case. The facility with which the peritoneum can be deflected from the presacral glands and tissue in this method of operation avoids the necessity, as in Kraske's operation, of frequently opening the peritoneum.

Dr. E. H. E. Stack showed a case of precocious menstruation. The girl was 5½ years, and mentally a little deficient. Periods began at 18 months, and have been nearly regular ever since, lasting three days and requiring seven diapers. The breasts began to develop about the same time, and have progressed since; now the diameter is 3 inches, and depth at nipple 1½ inches. The pelvis has a grown-up appearance, and, comparing its measurements with about twenty others of ages from 5 upwards, it conforms to the average of those of 12 years. The external genitals are not much abnormally developed; there is a little downy hair over them about ¼ inch long. She has been twice seen menstruating, and blood had the appearance of a normal period. There have been thirty to forty cases reported, and about twenty of these seem genuine and do not include cases beginning at 7 or 8 years or the bleeding once of new-born babes. They are very much alike. Average beginning was 2 years; most, but not all, had distinct growth of hair on pubes, but not in axilla. All had well-developed breasts; most were otherwise normal children; some seemed older than their years. Longest record is a child of 11, who began at 16 months. Only one case of very early puberty in a boy was found. No cause appears in the way of family history, climate, etc.—Dr. AUST LAWRENCE said that he would have liked to have had some information on the condition of the pelvic viscera as ascertained under chloroform or by rectal examination.—Mr. ROGER WILLIAMS regarded the condition as not so rare as was generally believed, and he had collected notes of over fifty cases. Nothing was more remarkable in the history of the development of the body than the great punctuality with which the different developmental events came off, although in exceptional cases a wide range of variability in this respect was not incompatible with health. Developmental anomalies with regard to time were quite as interesting and important as structural anomalies; and they were met with in respect to dentition and other conditions, besides sexual development. He thought many cases of this kind might be ascribed to reversion. Very few animals arrived at sexual maturity at such a late age as man; and among savages—where the waste of life was great—sexual maturity and reproduction ensued at much earlier periods than in modern communities. In this connection it was interesting to note that among the Hindoos—who were a branch of the same stock as ourselves—sexual maturity usually set in at an earlier age than with us, girls often attaining puberty at 8 years or even earlier. Premature sexual development was met with in boys as well as in girls, although most of the recorded cases were of the female sex. It might supervene at any time from birth to the normal age of puberty. A curious feature in many cases was that this precocity only manifested itself in respect to some of the constituent phenomena that go to make up sexual maturity. Thus in some cases the only evidence of it was mammary overgrowth; in others, menstruation; in others, undue development of the genitalia, etc. In most cases, however, the *ensemble* of the phenomena of sexual maturity was affected. Mr. Williams thought it might be inferred from the foregoing that the various phenomena which together constitute sexual maturity had been gradually integrated and co-ordinated, rather than that they had suddenly sprung into existence as a physiological unity. The internal sexual organs were often affected, as well as the external ones; for in necropsies on cases of this kind maturing Graafian follicles, corpora lutea, and recent ovarian cicatrices had been found with the pubescent uterus, ovaries, tubes, and pelvis; in short, there could be no doubt but that in these cases we had to do really with puberty. In support of this, he referred to cases of early pregnancy, at from $8\frac{3}{4}$ to 12 years, these young mothers having manifested signs of sexual precocity from earliest infancy. In one case of this kind the mother, aged 12, was impregnated by a boy of 14, and in due course she was delivered of a healthy child. Many cases of premature sexual development had been met with in children having intra-abdominal tumours, such as ovarian cystomata, sarcomata, etc., and adrenal tumours. In these cases the condition seemed to be caused, and the local congestion determined, by the presence of the tumour. In this connection it was interesting to recall the cases in which lactation had been excited—in young children and others—by repeated suction of the nipples, etc. Thus it seemed that sexual precocity might result from extrinsic as well as from intrinsic causes. —Dr. C. ELLIOTT stated that all cases of so-called premature menstruation should be carefully examined, as the discharge of blood from the vagina was not always from the uterus. Some years ago a young girl was brought to the Children's Hospital who had been seen by more than one medical man, and the mother was told by them that it was a case of premature menstruation. Not being satisfied with this diagnosis, he admitted the child into the Hospital and had the bladder examined. A hairpin was found with one point protruding through the urethra, and a phosphatic calculus had formed around it. His colleague, Mr. Norton, broke down the calculus, and removed the hairpin with some difficulty.

Dr. F. H. Edgeworth read a paper on the treatment of bronchitis, which will appear in a subsequent number of this *Journal*.

Mr. Arthur Prichard read notes on a case of hæmatometra in which, by strict antisepticism and slow draining of the tumour through the imperforate hymen, a cure resulted. The case was that of a girl aged 18, who first noticed a swelling in the lower part of the abdomen seven months before coming under observation. There was no pain at regular intervals. She had never menstruated. The vagina was occluded by a tense membrane, slightly bulging, to which an impulse could be communicated by pressure on the abdominal swelling. It was drained into sterilised wool pads by a small tube, and the catheter used when required. In three days the tumour, which before reached to 1 inch above the umbilicus, was almost gone. The discharge lasted for fifteen days. The vagina was subsequently dilated. The upper part was capacious, and had constituted part of the abdominal tumour. The os uteri would admit the tip of one's forefinger.—Dr. Aust Lawrence mentioned three cases of hæmatometra which he had seen. 1. Imperforate hymen, vagina and uterus distended, with retained menstrual blood. Treated by slowly draining through cannula and tubing into a bottle containing carbolic lotion. Perfect success. 2. Occluded os uteri, the result of *post-partum* sloughing of cervix uteri, the retained fluid caused uterine enlargement equal to a six months' pregnancy. Treated by slow draining through an opening made in position of normal os uteri, as case 1. Complete success. 3. Imperforate os uteri in one half of a bifid uterus, the other half menstruating normally. Treated as No. 2, but patient died from septic peritonitis, due to escape of menstrual blood into the abdomen, owing to rupture of the adherent and distended tube on that side. The symptoms of this were very indefinite.

---

*April 12th*, 1899.

Dr. J. E. Shaw in the Chair.

The Chairman showed a patient with peroneal type of muscular atrophy, which will be described in a later number of this *Journal*.—In connection with this case Dr. Michell Clarke showed photographs of the hands of a patient, aged 10, under his care eight or nine years ago. There was wasting of the small muscles of the hands and of the muscles of the forearms, and the fingers were contracted in flexion. The disease came on after one of the acute specific fevers. The wasting appeared to be stationary, and the patient after attending at the Hospital for some time was lost sight of. Her case was one of neuritic atrophy.—Dr. E. J. Cave mentioned the interesting pathological question raised by the fact that what appeared to be a primarily neuritic affection should be liable to occur in members of the same family, especially in regard to the part played by measles in the etiology of the condition. Such family groups were more familiar in primary muscular or spinal affections.

Dr. H. WALDO showed a patient with tubercular peritonitis treated by operation. He was brought to the Infirmary with a tubercular temperature, a tender and swollen abdomen, depending in the first instance upon tympanites and shortly afterwards on ascites. He was acutely ill, and Dr. Waldo thought an abdominal incision would give him the best chance. The most satisfactory results from this treatment occur in the ascitic variety.—Mr. W. H. HARSANT said that when the boy came under his care he seemed so ill and hectic, and was so rapidly losing weight and strength, that it was obvious he would not recover unless something were done. A small incision was made and 4½ pints of fluid were evacuated. The peritoneal cavity was then freely washed out with sterilised water, and the wound closed without drainage. Recovery was rapid and most satisfactory; and though it was too early yet to speak of a cure, it seemed as if this boy would probably remain well. It had been laid down by some observers that a cure could not be said to be assured until five years had elapsed. The cases most favourable for operation were those occurring in young patients with an acute history and where there is a free exudation of fluid, and all these circumstances were combined in the present case.—Dr. WALTER SWAYNE stated that he himself, some years ago, suffered from an affection which was diagnosed as tubercular peritonitis, with symptoms of abdominal distension, general wasting, slight ascites, and for a short time some evening rise of temperature. He recovered without operation, and was pleased to hear Mr. Harsant's statement that after five years the patient might be considered safe.—Mr. C. A. MORTON alluded to three cases under his own care in which ascites from tubercular peritonitis had been present, operation had been postponed, and in all the cases recovery had taken place without. He thought that surgeons had brought forward theories which, as Dr. Waldo had stated, were most unsatisfactory explanations of the improvement after laparotomy, because they had failed to recognise the fact that probably a large number of the cases operated on would have recovered without. Hawkins[1] had shown that 59 out of 100 cases of tubercular peritonitis, treated in the wards of St. Thomas's Hospital, got well without operation. But he thought that when it was necessary to evacuate the fluid on account of the pressure which it exerted, it was far safer to do so by incision than by tapping. A short time ago he had seen a lady suffering from ascites from tubercular peritonitis who had a few hours before been tapped, and only fæces had escaped. The area tapped had been quite dull, no doubt because the adherent bowel was at the time empty of gas. He thought the chance of adhesion of bowel to the anterior abdominal wall was so great in these cases that a free and careful incision was a much safer method of evacuating the fluid.—Dr. SHINGLETON SMITH mentioned an interesting history which had come to his knowledge. A patient in early life was supposed to be dying of tubercular peritonitis: she recovered, but some ten years later had spinal disease which caused considerable angular curvature: ten years later again she had hæmoptysis and indications of tubercular disease of lungs, together with a tubercular intercostal abscess. Death occurred some twenty-two years after the first indications of abdominal tuberculosis, but there was no return of the peritoneal symptoms.—Dr. J. O. SYMES said that in other collections of tubercular fluid it was noteworthy that evacuation was followed by rapid disappearance of tubercle bacilli. Thus pus aspirated from a tubercular hip-joint might be found crowded with bacilli, but if the discharge were examined twenty-four hours after opening the abscess the discovery of the bacilli

[1] *St. Thomas's Hosp. Rep.*, 1892, xx. 25.

would be a matter of great difficulty.—Mr. PAUL BUSH mentioned that in operating on these patients he endeavoured to admit as much air as possible into the peritoneal cavity, and that he had observed that the mere evacuation of fluid was often not sufficient to obtain a cure.—Dr. E. H. E. STACK remembered a case at St. Bartholomew's Hospital in a young man who, without ascites, had chronic intestinal obstruction which no medical treatment relieved. An incision was made through the abdominal wall, and the intestines were so matted that nothing further was done; but within a few days the obstruction was relieved, and in a few weeks the boy seemed quite well, and he was still well a year afterwards.—Dr. F. H. EDGEWORTH called attention to the statement of Fagge to the effect that the majority of cases of ascitic tubercular peritonitis are cured by the application of linimentum hydrargyri on lint to the skin of the abdomen.—Dr. MICHELL CLARKE said that he agreed with Mr. Morton that the majority of these patients got well from the immediate attack. Operation was unnecessary unless there was ascitic fluid, and then a certain time should be allowed for medical treatment to be carried out before operation was undertaken. If, however, an extreme amount of fluid was present so as to cause pain from distension, the abdomen should be opened without waiting. He mentioned a case in which a large quantity of ascitic fluid was absorbed as the result of extensive blistering of the abdominal wall, occurring from the application of a carbolic pad as a preliminary to operation. Tuberculous disease of the lungs, or persistent diarrhœa indicating intestinal ulceration, as a rule, contra-indicated operation. In these cases, as in a patient lately under his care, death might take place from obstruction of the intestine through adhesions or bands. Though the immediate prognosis of an attack of tuberculous peritonitis was good, the ultimate issue of the case was doubtful; three instances had occurred to him in the last three years in which the lungs became affected.—Mr. J. EWENS spoke in favour of blistering the abdomen with mercurial dressing in all cases of fluid in the abdomen; he had seen very good results. In some cases of ascitic fluid, when digitalis, squill, and other direct diuretics failed to reduce the swelling, small doses of mercury, given to the extent of affecting the gums, produced immediate diminution of the fluid.—Dr. R. C. LEONARD asked whether it was possible to exclude tubercular lesions of the intestine in cases of tubercular peritonitis, and whether if it was suspected operation was contra-indicated. He quoted Dr. Whitla's results with cod-liver oil in these cases.—Dr. C. ELLIOTT mentioned that in cases of tubercular peritonitis the secretion of urine was usually considerably diminished, and this fact he had found useful for diagnosis in obscure abdominal cases. For instance, he had once ventured on this ground to question the accuracy of his surgical colleagues in the case of a young woman who had been admitted for a supposed ovarian cyst. When operated upon, as he expected, simple peritoneal fluid was let out, and the patient made an excellent recovery. His treatment accordingly aimed at increasing the urinary excretion, and for this purpose he usually employed infusion of broom, combining it with iron, and the results were satisfactory.—Dr. WALDO, in reply, said that in cases where operation was not considered advisable he used lin. hydrargyri and a dry diet.

Dr. WALTER SWAYNE showed a specimen of diffuse sarcoma of the uterus with complete inversion. The patient, a nullipara, aged 17, was admitted to the Royal Infirmary with what looked like a piece of placenta protruding from an intact vaginal orifice. This was found to be immovable and to consist of part of the fundus covered with a placenta-like growth, a section from which showed that it was composed of mixed spindle- and round-celled sarcoma. The growth covered the whole of the body of the uterus, which was completely inverted and rreducible. A large vesico-vaginal fistula was present, the opening being about the size of a shilling. As the growth appeared to have infiltrated the surrounding parts, it was dealt with by removal of the growth from the fundus by scissors and curette. The patient improved markedly in health after this; but was readmitted three months later with recurrence of the growth, and again treated in the same way. Improvement again followed the removal; but four months later the patient was readmitted, and died on the third day after arrival of asthenia. While sarcoma of the uterus was rare, and inversion also rare, diffuse sarcoma of the fundus uteri was specially apt to cause inversion.—Dr. W. H. C. NEWNHAM said that inversion so complete as shown in this specimen must be extremely rare. He had only met with *five* cases of complete inversion: four were produced by the midwife pulling upon the cord; these were admitted into the Hospital, replaced, and all recovered. The other was produced by the dragging down of a large fibroid tumour, which was sloughing and projecting from the vulva. He passed the wire of an ecraseur round what he believed to be the neck of the tumour and removed it. On looking at the specimen, he found he had also removed the upper half of the uterus with the Fallopian tubes in addition to the tumour. He did not take any further operative steps, and strangely enough the patient recovered absolutely without a single bad symptom or rise of temperature.

Dr. E. H. E. STACK showed a specimen of retro-pharyngeal abscess. The child was 2½ years, and had been ill a fortnight before admission. He came in under Dr. Shaw and seemed to be a case of diphtheritic laryngitis. The fauces looked natural. There was so much recession that tracheotomy was decided on almost at once, but on the first stroke of the knife he cried with such a good voice that it seemed evident there was no laryngitis. Exploring with the finger, a retro-pharyngeal abscess was felt and opened from behind the sterno-mastoid, several ounces of pus were evacuated, and very soon the breathing was relieved and he could swallow; but in a day or two he died, not having been able to recover the serious dose of septic infection he had received. The specimen shows no evidence of tubercle of the spine or other evidence of the cause of the abscess.

Dr. BERTRAM ROGERS read a paper on a case of thrombosis of the portal vein (*see* page 107 of the current number).

Mr. PAUL BUSH read a paper on some peculiar operation cases in lunatics, which will be published in a subsequent number.

---

*May 10th*, 1899.

Dr. A. J. HARRISON in the Chair.

Mr. PAUL BUSH showed a boy with a marked displacement of the right scapula, which was raised about 3½ inches and rotated, the lower angle of the bone being situated over the spine of the vertebræ; there was no winged condition of the scapula. He considered the case was one of spasm of the trapezius muscle. A clear skiagram showed that the upper vertebræ were drawn over to the affected side.

Mr. J. LACY FIRTH showed a case of cervical ribs present on both sides, but more conspicuous on the left side. There were some signs of pressure on the brachial plexus and the subclavian artery on the left side.—Mr. ROGER WILLIAMS said a few years ago Prof. Debierre read a paper at the International Medical Congress on "The Human Thorax: is it regressing?"[1] In support of his views, he referred to man's lost cervical and abdominal ribs, regarding specimens such as that shown this evening as due to reversion. The first and the two last ribs were normally of stunted and imperfect formation, indicative of tendency to disappear; and sometimes the first rib had no connection with the sternum, or it was represented only by a fibrous band. The chest muscles, especially the intercostals and those over the ribs, presented signs of regression in the great abundance of their tendinous structures. Similarly the thoracic (dorsal) nerves were seldom complete, for certain of them were wanting in 81 out of 100 cases; and nerve roots were defective in many cases. Mr. Williams had been specially interested in this subject, because his studies in polymastia had independently led him to a similar conclusion, he having shown that man had lost six pairs of mammary glands from the front of the thorax, three from above and three from below the normal pair, and traces of these lost mammæ occasionally reappeared owing to reversion. From the great thickening of the abnormal rib structure in the case exhibited, and from the fact that pressure-symptoms had been noticeable of late, he thought the redundant rib was growing in a very irregular manner; and in many cases irregular osseous growths, like exostoses, had resulted from the abnormal evolution of these formations.

Dr. W. H. C. NEWNHAM showed two specimens from the same patient —a large ovarian cyst, which was pressing on the bladder and rectum (being wedged down in the pouch of Douglas), and a fibroid uterus. The patient, aged 40, had one child thirteen years ago, and was sent by Dr. E. Walker for complete obstruction to micturition and partial obstruction to defecation. The ovarian tumour was distinctly felt in the pouch of Douglas. On April 20th he removed the cyst, also the fibroid uterus, by the operation of intra-peritoneal hysterectomy. The right ovary he left behind, it being apparently healthy. The woman recovered without a bad symptom, and was walking about the ward eighteen days after the operation.—Mr. C. A. MORTON said that evidently the ovarian cyst, which he understood had been supposed to be a myoma before operation, was the cause of the pressure-symptoms, and he did not think any need had been shown for performing hysterectomy. He thought that the mere fact that the abdomen had been opened was not sufficient reason, for the great majority of myomata never caused any trouble at all, and unless they grew very large, or caused pressure-symptoms or serious hemorrhage, there was no need to interfere with them. He quoted from Mr. Bland Sutton's recent papers advising removal of every myoma just as we remove every ovarian cyst, and he contended that a surgeon who did this would be likely to have a run of simple cases, and that his results by the intra-peritoneal method could not be compared with those of surgeons who only operated on very large tumours or those causing pressure-symptoms or serious hemorrhage by the extra-peritoneal method; and he referred to the mortality of one such operator who had had a more successful run of cases by the extra-peritoneal method than Mr. Bland Sutton by the intra-peritoneal. He also pointed out that the mortality in Bland Sutton's twenty-eight cases was 7 per cent., and that this was a high mortality for ovariotomy at the present time, and that therefore the mortality of the intra-peritoneal method

[1] *Atti d. xi. Cong. med. internaz.*, 1894, ii. Anat., 12.

could not be said to be the same as that of ovariotomy. Mr. Morton also referred to the dreadful picture of the results of the extra-peritoneal method drawn by Dr. Newnham in his recent paper in the *Journal*,[1] and said he ventured to think it was altogether an exaggeration. He asked, what evidence was there that dragging on a pedicle had ever caused peritonitis? and denied that fetid pus was discharged from the pedicle, or that there was a danger of pus leaking into the peritoneal cavity and causing peritonitis. He asked if there was no possible risk of peritonitis by the intra-peritoneal method from septic infection from the interior of the uterus, and if one could ignore the risk of injury to the ureters in the ligation of the uterine arteries? He did not wish to plead for the extra-peritoneal method in all cases, but mentioned some conditions in which it might be an advantage, and referred to one of his own cases treated by that method, with success, in which the tumour was very large—reaching up to the costal margin,—and in which the operation was rendered difficult by intimate adhesions to the bladder, and an expansion of the broad ligaments so that he was glad to use the serre-nœud.—Dr. JAMES SWAIN said that it was an easy matter to criticise another man's work when the specimen appeared on a plate, but the operator could best determine the right course of action to pursue, for at the time of operation we might consider it desirable to do that which might afterwards appear to others to be unnecessary. Speaking of the removal of fibroid tumours of the uterus in the abstract, he thought that the surgeon who performed hysterectomy in such cases should always be able to give a good reason for his action apart from the mere presence of a tumour. With reference to the battle which was being waged between the extra-peritoneal and the intra-peritoneal treatment of the pedicle in hystero-myomectomy, he felt that statistics were not altogether trustworthy, especially as such men as Bland Sutton apparently favoured the removal of all myo-fibromata, apart from their size or symptoms. He thought that of more value than statistics was the significant fact that most operators all over the world were now adopting the intra-peritoneal, or rather the retro-peritoneal, method. Thanks to the use of antiseptics, the employment of the clamp in extra-peritoneal hysterectomy had not been attended with such disastrous results as had accompanied the use of the clamp in the early days of ovariotomy. The extra-peritoneal method had been most successful, and he had not seen any of the unpleasant accompaniments which had been said to follow this mode of treatment. Each method appeared to be attended with good results, and he thought that the balance in favour of the intra-peritoneal method was to be looked for not so much in a diminished mortality as in greater precision in operating, a more speedy convalescence, and a lessened tendency to ventral hernia. He had recently adopted the retro-peritoneal method, and would continue to use it in suitable cases, for he regarded it as the operation of the future. At the same time, he had had such successful results with the clamp that he did not think it should be entirely discarded, for he thought there were cases where this method might advantageously be adopted, though not as routine treatment. The question was in a transition stage, and he declined to pin the whole of his faith to a pedicle clamp with the "extra-peritonealists," or to cast it free in the abdomen with the "intra-peritonealists." Our duty was to get the patient well by the best means at our command, and all would prefer to do a successful operation by what might be considered an incomplete technique, rather than to perform a surgically

[1] 1899 xvii. 8.

perfect operation resulting in the death of the patient.—Dr. Walter Swayne was of opinion that it was impossible to say from seeing pathological specimens after operation whether operation was indicated in a particular case or not. It is a matter of experience that the most serious symptoms are not always produced by the largest tumours. He had now under his care a patient suffering from a fibroid which reached nearly to the ensiform cartilage, and was accompanied by profuse hemorrhage; by treatment and natural processes (the menopause) the hemorrhage and general symptoms soon ceased and the tumour retrogressed. The patient refused operation, perhaps wisely. At the same time small tumours produce occasionally very severe symptoms, and in any case when pain produces such disturbance that the patient is prevented from earning her living and her life is made a burden to her, then operative removal is imperative. He thought that if Dr. Newnham had not removed the uterus and the patient's symptoms had been unrelieved, his position as regards the patient would not have been a pleasant one. It appeared to him that the question of what was to be done at the operation must be left to the operator, and could not be settled by the inspection of a specimen previously removed by those unacquainted with the particular symptoms present. —Mr. T. Carwardine spoke in favour of the intra-peritoneal method of treatment of the pedicle in hysterectomy. With regard, however, to the extra-peritoneal treatment of the stump, any moisture and suppuration in the stump are usually due to faulty procedure and after-treatment.—Mr. Roger Williams thought the term "hysterectomy" ought to be restricted to total extirpation of the uterus. The application of this term to all sorts of other operations comprising partial removal of the uterus tended to produce confusion. Dr. Newnham's case was one of amputation of the myomatous corpus. The small size of the tumours, the fact that the largest of them was connected with the fundus by but a slender pedicle, and that the other two tumours were of sub-peritoneal evolution, led him to ask the author why he had preferred amputation of the uterus to myomectomy, the procedure which Mr. Williams would have preferred, as it would have left the uterus intact, and was safe and efficient. Mr. Williams said it was misleading to compare statistics as to mortality, unless like things were compared with like things. The class of cases a surgeon had to deal with who undertook to remove every myoma, as soon as its presence could be recognised, was totally different from the class of cases that had to be dealt with by those who only operated when urgent symptoms arose. The former had to deal only with small tumours without extensive adhesions, and the mortality from such operations was from 5 to 10 per cent. In these early cases the condition of the uterus and its adnexa differed but little from the normal. In the other class of cases, however, the tumours were often of great size, very vascular, extensively adherent, inflamed and otherwise complicated, so that equally good results could not fairly be anticipated. In most of the former class of cases Mr. Williams thought operation unnecessary and unjustifiable. Hysterectomy, as a conservative operation, was a remedy worse than the disease it was meant to cure.—Dr. Newnham, in reply, said it was a matter of opinion as to whether this was the right operation for this patient. She was complaining, in addition to her other troubles, that she had to keep her bed for ten days every month on account of the uterine hemorrhage. Supposing he had left these fibroid tumours, would she not rightly have returned complaining he had not cured her of her ailments. He did not think it possible that the ovarian cyst was the cause of the hemorrhage. In all probability the myomata would grow to a large size in a woman whose age is only 40. He did not say that it is necessary to remove every myoma, for he had dozens of women attending as out-patients, and not one of them did he propose to operate on, because they had no serious symptoms. Neither did he say that every case that is undertaken must be done by the intra-peritoneal method, each case should be decided on its own merits; but he did think that intra-peritoneal hysterectomy is the operation of the future. He thought that if he had attempted to enucleate these fibroids from the uterus, as Mr. Williams suggested, the hemorrhage would have been more profuse, and, in his opinion, the danger of the operation would be greater than that of intra-peritoneal hysterectomy.

Dr. T. Fisher showed the following specimens:—(1) A pancreas containing several calculi from a man, aged 58, who died in the Bristol Royal Infirmary of senile gangrene. Apparently the calculi had given rise to no symptoms. (2) One of fibroid myocarditis, from a young man, aged 18, who had died suddenly while at work. There was disease of the aortic valves, but no affection of the coronary arteries. The chief points of interest were—firstly, that the fibroid disease was with very little doubt due to rheumatism; and, secondly, that, as so commonly occurs in cases of fibroid disease in later life, the patient died suddenly. (3) A specimen of broncho-pneumonia breaking down into cavities. Dr. Fisher thought that many cases of so-called dilated bronchial tubes are really the sequence of actual lung-destruction consequent upon broncho-pneumonia. (4) Specimens of sarcoma of the cæcum and of the ileum.—Mr. Roger Williams said much interest attached to examples of malignant disease of the cæcum and small intestine, on account of their variety. Of 1,066 primary sarcomata tabulated by him, there was not a single instance of sarcoma of the small intestine. In 1892 Madelung reported three cases, and gave references to eleven others. Mermet, in 1896, reported a case of sarcoma of the jejunum in a woman, aged 32, who died the day after resection of the diseased bowel. She suffered from pain and diarrhœa, but not from obstruction. Twenty-three other cases were cited. Since then a few others had been reported. Several cases had been met with in quite young infants. In Stern's patient—an infant only a few days old—the disease presented as polypoid angio-sarcoma of the ileum, which caused death by intestinal obstruction. He thought these polypoid sarcomata generally arose from congenital adenomata of the intestinal mucosa.—Dr. P. Watson Williams remarked that dilatation of the bronchial tubes, due to paresis of the muscular coat, was usual in bronchitis of the smaller tubes and broncho-pneumonia, and almost invariably found in fatal cases, and in this way the great majority of dilatations of bronchial tubes originated. In a comparatively few cases, as in the specimen shown by Dr. Fisher, the dilatation was due to the action of various micro-organisms leading to erosion or destruction of the bronchial wall and the neighbouring pulmonary tissue.

Dr. Bertram Rogers showed two microscopic sections of the liver of a child. The patient had been for about eight weeks in the Hospital, when she developed a high temperature and died. The only symptoms were fever, an enlarged liver and slight jaundice. The liver *post mortem* presented a very mottled appearance, parts being fatty and parts very deeply congested, and in places a deep red from hemorrhages. Under the microscope, there were large patches where the liver substance seemed to have disappeared, leaving only a fine reticulum of connective tissue. There were extensive small hemorrhages into the substance of the liver.—Dr. T. Fisher, in connection with this case, showed a drawing he had made of a very similar condition he had found in a case of heart disease.

Dr. E. H. E. Stack showed the following kidney specimens:—Two showed abscesses following bladder disease, one acute, the patient having only lived nine days after he broke his back. Two cases show hemorrhage in the pelvis of the kidney, the first from a case of purpura, under Mr. Munro Smith, in which the source could not be discovered. The patient was a man who had epistaxis, melæna and hæmaturia, as well as extensive hemorrhages in skin and muscles. Microscopically, the blood was normal. The second is from a case presented to the Infirmary by St. Bartholomew's Hospital—it is a kidney from bubonic plague. The two specimens are very much alike, the pelvis and ureter being filled with blood-clot. Two cases show adenoma of the kidney—one a single one, and the other multiple small ones. Two cases show hydronephrosis—one from stone impacted in the ureter, the other much larger, and for which, although the ureter was found by operation to be quite occluded, no cause could be discovered. Two cases show dilatation of the ureters, both from babies. No cause can be assigned for this, as there is no obstruction to the flow of fluid from ureter to bladder. One case of small multiple sarcomata from a child who died of diarrhœa, no other growth found. The last case is one of thrombosis of the renal vein. The child died of infantile diarrhœa. It is interesting in the light of recent bacteriological research on the streptococcus nature of this variety of diarrhœa. Dr. Symes grew from the clot many colonies of streptococci.

Dr. W. H. C. Newnham read a paper on "Removal of one Ovary—with Cases." The record of the following cases may be of interest, as they include some women who have been under treatment for long periods (occasionally extending to years) by pessaries and various intra-uterine medication without relief. He simply included cases of painful prolapsed or inflamed ovary on one side. The operation for the removal of the diseased organ is simple, takes a very short time, and in the cases given below appears to have relieved all the symptoms and changed the patient from a chronic invalid to a worker.

(1) S. J., aged 26, laundry-maid, single. Has suffered with pelvic discomfort for eleven years,—much worse the last six, especially during catamenia. The pain was always on the left side. She had been previously treated with a pessary after some intra-uterine application. On April 7th, 1892, he removed the ovary, enlarged and deeply grooved apparently by the edge of the Hodge's pessary. She left the Hospital well on May 17th, and continues in good health.

(2) K. T., aged 25, unmarried, was admitted complaining of intense pain on the right side and down the right groin. He removed a large cystic and prolapsed ovary of the right side on August 10th, 1892, and the patient left the Hospital well on September 12th.

(3) A. B., aged 28, unmarried, came with a history of gonorrhœa some six years previously. She complained of intense pain in the right ovarian region. He removed a large inflamed right ovary on March 19th, 1898. She left the Hospital well on April 27th, 1898.

(4) F. O., aged 26, married. Had been ill for three years, complaining of dyspareunia and menorrhagia. Has had no children. He removed an intensely painful prolapsed ovary on the right side on April 21st, 1898, and patient left the Hospital on May 28th, 1898, quite well. She is now six months pregnant.

(5) M. L., aged 32, waitress, unmarried, with a history of gonorrhœa. Nine years ago she had first child. For the last eight years has been constantly in bed with some inflammatory attacks in the lower part of the abdomen. *Per vaginam* on right side was felt a swelling, about the size of a small orange, which was acutely painful. He removed the right ovary on December 20th, 1898; it was very large and congested, and the tube itself was much swollen. For the first three days after the operation patient was very sick, in great pain, and distended to an uncomfortable degree. This all subsided under appropriate treatment, and patient left the Hospital quite well on January 17th. She now appears to be in perfect health.

(6) A. J., aged 32, married ten years, has had one child. Has had pain in left groin for twelve years. He removed a large tender and prolapsed ovary on the left side on February 20th, 1899, and she left the Hospital quite well on March 24th, 1899.

(7) M. R., aged 25, single, came with a history of localised peritonitis. On examination he found a large inflamed and apparently adherent ovary on left side This he removed August 20th, 1898, with some difficulty, as the adhesions were very dense all around. Patient, however, recovered without a bad symptom, and left the Hospital on September 28th, 1898, quite well.

(8) J. I., aged 28, had suffered from abdominal pain and discomfort for four years in the region of the left ovary. She is a married woman and has had three children. He removed a cystic ovary from the left side on April 12th, 1899, and patient is now well.

Dr. Walter Swayne considered that there were many useful points to be noticed in connection with the series of cases. The first case illustrated very well the importance of a correct use of pessaries; it should be an axiom that a pessary should never be used if it pressed on solid visceral structures, *e.g.* uterus or ovary; intra-uterine applications also should be avoided, except after free dilatation and provision for drainage. In any case where symptoms of one-sided tenderness on pressure, backache, dysmenorrhœa, sterility, and dyspareunia were present, the condition of the ovary, if felt to be enlarged, should be investigated by operation, unless the symptoms were alleviated by rest and local treatment; in a large number of these cases, even when temporary alleviation took place, operation was ultimately necessary. He mentioned a case in which he removed the left ovary from a patient suffering from the above symptoms. On bringing the ovary into the wound it was seen to be cystic: the cysts were punctured, and the fluid contents evacuated; but as the diseased portion proved more than one-half of the ovarian substance, instead of removing the cystic portion from the ovary and returning the remains of the latter after suture, he removed the entire ovary. The patient was able to go home on the twenty-first day after operation, perfectly well. The ovary at present might be seen in the museum.

J. Paul Bush, *Hon. Sec.*

---

